# Sleep Quality, Insomnia Symptoms, and Depressive Symptomatology among Italian University Students before and during the Covid-19 Lockdown

**DOI:** 10.3390/ijerph182413346

**Published:** 2021-12-18

**Authors:** Lorenzo Viselli, Federico Salfi, Aurora D’Atri, Giulia Amicucci, Michele Ferrara

**Affiliations:** 1 Department of Biotechnological and Applied Clinical Sciences, University of L’Aquila, 67100 L’Aquila, Italy; lorenzo.viselli@graduate.univaq.it (L.V.); federico.salfi@graduate.univaq.it (F.S.); aurora.datri@univaq.it (A.D.); giulia.amicucci@uniroma1.it (G.A.); 2 Department of Psychology, Sapienza University of Rome, 00185 Rome, Italy

**Keywords:** COVID-19 lockdown, university students, sleep quality, insomnia, depression

## Abstract

The COVID-19 pandemic led world authorities to adopt extraordinary measures to counteract the spread of the virus. The Italian government established a national lockdown from 9 March to 3 May 2020, forcing people in their homes and imposing social distancing. During the pandemic emergency, university students emerged as a vulnerable category. Indeed, higher rates of sleep problems and mental disorders were reported in this population. However, these outcomes were derived from cross-sectional investigations adopting retrospective assessments. Retrospective evaluations suffer from different biases, putatively leading to erroneous outcomes. To overcome this limitation, we adopted a between-subject approach comparing a sample of 240 Italian undergraduate university students assessed in 2016 (mean age ± standard deviation, 20.39 ± 1.42, range 18–25; 80.42% females), with an age/gender-matched sample of university students assessed during the third week of lockdown in Spring 2020. We evaluated sleep quality, insomnia symptoms, and depressive symptomatology using validated questionnaires. We found worse sleep quality, a delayed bedtime, and more severe insomnia and depression symptoms in the students sampled under COVID-19 restrictive measures. We suggest paying special attention to this at-risk population during the current pandemic emergency and applying preventive and supportive interventions to limit the exacerbation of sleep and psychological problems.

## 1. Introduction

At the end of November 2019, the novel coronavirus disease 2019 (COVID-19) started to circulate in China, before spreading worldwide. On 11 March 2020, the World Health Organization (WHO) declared the COVID-19 as a global pandemic. Italy was the first western hotspot of COVID-19. On 9 March 2020, the Italian government imposed a national lockdown. The restrictive measures forced people into their homes, imposing social distancing. Most working activities were suspended or switched to remote modality for the entire lockdown period, and only essential stores remained open (e.g., grocery; pharmacy). Universities interdicted in-person activities, preferring the online modality for lessons and exams. Moreover, people were forbidden to meet relatives and friends, and, at least during the first month of lockdown, going out for sport activities was prohibited. These measures were applied successfully to contain the spread of the infection [1], but they inevitably led to negative consequences for mental health [2].

High levels of depression, anxiety, and stress were highlighted during the COVID-19 lockdown among the general population [3,4]. Recent evidence has suggested that people perceived this situation as a traumatic stressor [5,6]. Indeed, the experience of home confinement or COVID-19 infection was associated with higher rates of post-traumatic stress disorder (PTSD) [7,8]. A heavily affected behavior among the general population was sleep [9]. Specifically, Italian citizens showed a worsening of their sleep quality and an exacerbation of insomnia symptoms during the COVID-19 lockdown [10,11].

Some population categories demonstrated a greater susceptibility to the effects of the restrictive measures. The university students emerged as one of the most affected categories [12,13]. Elmer et al. [14] reported a worsening in depressive and anxiety symptoms, and higher levels of stress and loneliness among the university student population during the restrictive measures, compared with pre-lockdown assessments. Moreover, recent cross-sectional investigations focused on students have showed high rates of depression, anxiety, stress, and suicidal thoughts during the pandemic emergency worldwide [14,15]. These findings are consistent with several Italian reports [6,12,16]. Worldwide, the confinement measures also seemed to have a detrimental effect on sleep in the university students [13,17]. This evidence has been confirmed by cross-sectional investigations among the Italian population [6,12].

In order to better understand the effects of home confinement on sleep and mental health among this specific population, it would be useful to have a direct comparison between data collected during the lockdown and the pre-outbreak period. However, current literature focused on students that adopts a longitudinal approach using a pre-outbreak baseline is limited [13,14,18,19]. For the Italian university student population, only one study had pre-outbreak data on mental health [16]. However, this investigation did not assess possible changes in sleep variables. On the other hand, several cross-sectional studies employed a retrospective approach referred to the pre-pandemic period to investigate the impact of restraining measures, due to the COVID-19 outbreak [12,20,21]. This methodology could be questionable as the retrospective symptom evaluations are frequently biased, and subjects tend to overestimate the current symptomatology [22,23]. Moreover, depression and anxiety affect the accuracy of the retrospective recalls [23]. These biases were confirmed by the findings of Gao and Scullin [24], who adopted both retrospective and longitudinal approaches and found a worsening of sleep quality only in the first case. One way to partially overcome the limitations of retrospective studies is to compare the data obtained during the pandemic with those previously collected on a different sample from the same population. Although useful in this unprecedented period, this study typology is limited [14,25], and even absent among the Italian student population.

Based on the above-mentioned evidence, we evaluated the effects of the lockdown on sleep quality/patterns, insomnia symptoms, and depression symptomatology in undergraduate students by comparing data from a previous study [26], collected in October 2016, with data from an age/gender-matched sample of undergraduate university students collected in the first week of our investigation held during the lockdown period of Spring 2020 (25–31 March 2020) [11].

Considering the peculiar and vulnerable life condition of the university students under restrictive measures, we hypothesized that students assessed during lockdown showed worse sleep quality and a higher level of insomnia and depression symptoms compared to students assessed during the pre-pandemic period. Finally, the lack of social impositions due to the restrictive measures could lead undergraduate students to follow their well-documented evening circadian preference [27,28]. Therefore, we hypothesized that there would be a delayed bedtime and get up time in the sample evaluated during the lockdown.

## 2. Materials and Methods

### 2.1. Participants and Procedure

In the present study, data derived from two different samples of undergraduate university students are reported. The first one (pre-pandemic group) consisted of a sample of 240 Italian students (mean age ± standard deviation, 20.39 ± 1.42, range 18–25; 80.42% females) who participated in a data collection held from 6 to 11 October 2016 at the University of L’Aquila [26]. The second sample (lockdown group) comprised a total of 240 Italian students evaluated during the third week of home confinement period (25–31 March 2020), matched for age and gender with the pre-pandemic group. Specifically, the lockdown group was randomly selected from our nationwide dataset [11], using a custom-made MATLAB script (MATLAB R2021a, The MathWorks Inc., Natick, MA, USA). We chose to match for age and gender as the two demographic factors are associated with different sleep characteristics [29].

In both the surveys, we collected demographic factors (age, gender), and we evaluated sleep quality, insomnia symptoms, and depression symptoms using the Pittsburgh Sleep Quality Index (PSQI) [30], the Insomnia Severity Index (ISI) [31], and the Beck Depression Inventory-Second Edition (BDI-II) [32], respectively. The PSQI is a validated 19-item questionnaire used to assess sleep quality covering seven different dimensions (subjective sleep quality, sleep latency, sleep duration, habitual sleep efficiency, sleep disturbance, sleep medication, daytime dysfunction). Each element is scored between 0 and 3, giving rise to a total sleep quality score. Higher scores (range 0–21) reflect more severe sleep difficulties [33]. The ISI is a validated 7-item clinical tool to evaluate the severity of insomnia symptoms. A higher ISI score indicates worse insomnia symptoms [34]. The BDI-II is a 21-item questionnaire used to evaluate clinical depression symptoms. A higher score (range, 0–63) reflects more severe depression symptoms [32].

Both the studies [11,26] were approved by the institutional review board of the University of L’Aquila (protocol n. 23038/2016; protocol n. 43066/2020, respectively) and were performed according to the principles established by the Declaration of Helsinki.

### 2.2. Statistical Analysis 

We performed independent sample *t*-tests comparing the PSQI, ISI, and BDI-II scores of the two student groups (pre-pandemic group, lockdown group) to evaluate putative differences in sleep quality, insomnia, and depression symptoms due to the lockdown period. The same analysis was performed for each sub-component of the PSQI (subjective sleep quality; sleep latency; sleep duration; habitual sleep efficiency; sleep disturbance; sleep medication; daytime dysfunction) to provide a fine-grained overview of the sleep differences between the two conditions. We also extracted from the PSQI questionnaire two crucial variables, such as bedtime (hh:mm) and get up time (hh:mm) to evaluate possible differences in sleep schedule. Independent sample *t*-test analyses were applied to the above-mentioned sleep pattern variables (bedtime, get up time).

Furthermore, we carried out an analysis of variance (ANOVA) on PSQI, ISI, and BDI-II scores, with gender (male, female) and condition (pre-pandemic, lockdown) as two level between-subjects factors. Such analysis was performed both to assess possible gender effects on sleep quality, insomnia, and depression symptoms and to explore putative interactions between the two factors (“gender” × “condition”). 

## 3. Results

The results of the comparisons between pre-pandemic and lockdown groups on the PSQI, ISI, and BDI-II scores, PSQI sub-components, and bedtime and get up time are summarized in Table 1.

**Figure 1:**
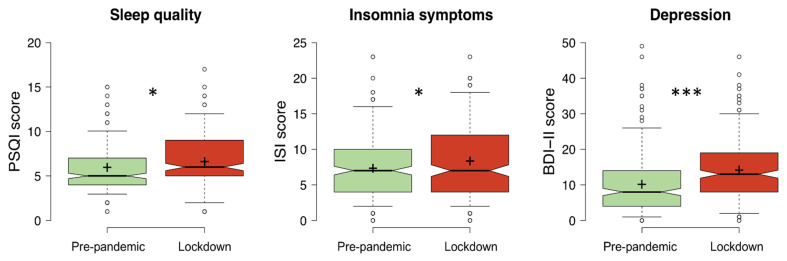
Sleep quality (PSQI), severity of insomnia (ISI), and depression symptoms (BDI-II) of the students in pre-pandemic group (assessed in 2016) and the group of students evaluated during the COVID-19 lockdown. Center lines show the medians; box limits indicate the 25th and 75th percentiles; whiskers extend to 5th and 95th percentiles, outliers are represented by dots; crosses represent sample means. Significant differences between groups of students (pre-pandemic, lockdown) are indicated with asterisks (* *p* < 0.05, *** *p* < 0.001). Abbreviations: PSQI, Pittsburgh Sleep Quality Index; ISI, Insomnia Severity Index; BDI-II, Beck Depression Inventory-Second Edition.

**Figure 2:**
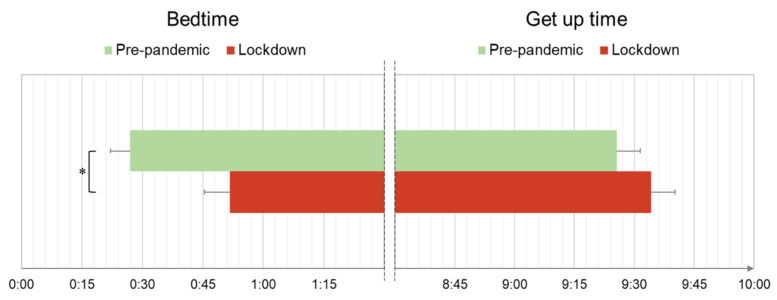
Sleep patterns of the two student samples (pre-pandemic, lockdown). Means and standard errors of bedtime and get up time and significance of statistical comparisons are reported (* *p* < 0.05).

As shown in Figure 1, the students under lockdown reported poorer sleep quality, and more severe insomnia and depression symptoms than the pre-pandemic group. Moreover, the lockdown group displayed a delayed bedtime compared to the pre-pandemic group (Figure 2), but no differences in get up time were obtained.

In addition, students assessed under lockdown reported significantly poorer subjective sleep quality and greater daytime dysfunction than students evaluated during the pre-pandemic period.

Finally, the female gender emerged as the most compromised in sleep quality, insomnia, and depression symptoms, as the “gender” factor was significant in all the analyses (PSQI: F_1,476_ = 7.28, *p* = 0.007; ISI: F_1,476_ = 9.40, *p* = 0.002; BDI: F_1,476_ = 15.91, *p* < 0.001). However, no “gender” x “condition” interaction was significant for the assessed variables (PSQI: F_1,476_ = 0.36, *p* = 0.55; ISI: F_1,476_ = 1.78, *p* = 0.18; BDI: F_1,476_ = 3.14, *p* = 0.08).

## 4. Discussion

In the present study, we showed significantly worse sleep quality and more severe insomnia and depression symptoms among undergraduate university students during the COVID-19 lockdown compared to an age/gender-matched sample of students assessed four years before the pandemic period. The analysis of the PSQI sub-components identified daytime dysfunction and subjective sleep quality as the most affected sleep quality facets during the lockdown. Moreover, we reported that restraining measures negatively affected sleep quality, insomnia, and depression symptoms of male and female students in the same way. However, women reported higher scores in all the questionnaires regardless of the assessment period. These findings are not surprising since females show more severe sleep problems and depression symptoms both before [30,35,36] and during the COVID-19 lockdown [37]. As hypothesized, we also found a significantly delayed bedtime in the sample evaluated during the home confinement. On the other hand, we did not find any significant difference in get up time. So, our second hypothesis is only partially confirmed.

Several cross-sectional investigations reported a high level of insomnia symptoms and poor sleep quality in the university student population [10,12,38]. Moreover, a longitudinal study highlighted worsened sleep quality among students during the COVID-19 lockdown [17], pointing out a negative effect of the restrictive measures. To the best of our knowledge, before the current study, there was only one study [25] that assessed insomnia symptoms using validated questionnaires both before and during home confinement among independent samples of undergraduate university students. Benham [25] did not find any significant difference in insomnia symptoms, while we highlighted more severe insomnia symptomatology during the lockdown. However, the data from Benham [25] were collected in the Southwestern United States, i.e., a region where the number of deaths was substantially lower than in Italy at the moment of the assessment, a factor that could explain the different results [39,40]. Furthermore, data collection was not performed during a period of restrictive measures, but during the spread of the virus *before* the lockdown.

High daytime dysfunction in students during lockdown has been repeatedly documented [25,41,42], together with low subjective sleep quality [41,42]. For example, in Saadeh and colleagues [42], about half of the sample reported both difficulties staying awake during the day and poor subjective sleep quality. Moreover, in the same study, up to 80% of the respondents did not have enough enthusiasm for daily activities, showing an altered daily functioning. Similarly, Benham and collaborators [25] highlighted a worsening in diurnal functionality relative to a pre-pandemic condition. Daytime sleepiness experienced during lockdown may have played a role in the students’ perception of poor sleep quality. Furthermore, daytime functioning may have been affected by different factors, such as stress [43,44], loneliness [45], and worries about COVID-19 [46]. The current literature also showed a delayed bedtime and get up time during home confinement relative to the pre-pandemic period among university students [12,13]. However, the results of our study supported only the first assumption, pointing out a delayed bedtime in the student population. University students typically show an evening chronotype, preferring to go to sleep and wake up later [27,28]. Consequently, we hypothesized that the students assessed during the lockdown, free from university (i.e., lessons) and social demands, aligned their sleep rhythm more strictly with their biological rhythm, moving bedtime forward. On the other hand, the lack of a significant effect on get up time in our lockdown group could be attributed to seasonal influences on wakeup time, due to the different assessment period in the two samples, as spring season is associated with an earlier wake up time [47].

As far as depressive symptomatology is concerned, the literature highlighted high rates of students who reported depression symptoms during home confinement worldwide [6,14,15,48]. Furthermore, several longitudinal studies indicated a putative role of restrictive measures in exacerbating the depression symptoms among the university population [12,16,18,19]. Therefore, our results are consistent with the above-mentioned literature.

The restrictive measures obligated people to spend all days in their homes without social interaction and face-to-face peer contact. The university years coincide with a period of life characterized by important personal life events where social interactions are fundamental [49]. Poor social life is associated with negative health outcomes [50,51,52], and a change in social activities is considered an important stressor in this population [53]. During the lockdown, an additional detrimental factor was the concern about COVID-19, which negatively impacted mental well-being [46]. Based on this evidence, it is not surprising that recent studies showed that the collapse of social interactions during the pandemic emergency, and the worries (e.g., about close friends, or COVID-19), impacted the student’s mental health and sleep [14,46,54,55]. 

Students’ problems could also be caused by the influence of the pandemic emergency on their future carrier. During this unexpected situation, universities switched exams and lessons to online modalities. However, online education could have negatively affected student sleep and mental well-being [56]. Online learning has been proposed to exacerbate depression and anxiety symptoms [57]. Together with the desire to maintain social interactions and to counteract boredom, online learning led students to increase the use of electronic devices drastically [48,58]. Remarkably, the increased use of electronic devices before bedtime during lockdown was associated with worsened sleep quality and insomnia symptoms, as well as altered sleep-wake patterns [59]. This evidence was confirmed also during the second contagion wave during Winter 2020 [60].

Moreover, a good amount of physical activity is known to positively affect sleep and mental health [61,62]. However, during the home confinement students showed decreased physical activity and increased time spent sitting during the day [63,64,65,66]. The above-mentioned factors were associated with inadequate sleep and the development of psychological and health problems among the student population [61,67]. Interestingly, Caldwell and colleagues [68] highlighted a negative influence of the COVID-19 lockdown on dietary intake. Indeed, energy intake increased in university students during the lockdown, especially due to increased snacking frequencies [69]. Considering the consumption of fatty/sugary foods is associated with sleep problems in students populations [70,71], it is plausible that the variation in eating habits also influenced sleep quality during the lockdown.

Furthermore, younger people generally showed a tendency to evening chronotype [27]. Eveningness is associated with mental health problems and sleep disorders [27], and this relationship was also confirmed during the pandemic scenario [11]. In addition, younger people typically have the lowest level of resilience [72], and this feature was confirmed during the pandemic [73]. Rossi and colleagues [73] highlighted that age moderated the mediation role of resilience in the relationship between pandemic-associated stressful experience and depression, anxiety, and perceived stress. Overall, this evidence supported the idea that problems of young people may be attributable also to the lower resilience attitude [74]. 

Some limitations of the present study should be reported. We acknowledge that the non-probabilistic sampling technique adopted, and the use of self-report questionnaires may limit the generalization of our findings. Furthermore, we did not assess the socio-economic status of participants, their study fields, data about physical activity and dietary intake, or the presence of concomitant disease/treatment that could interfere with sleep problems. Moreover, a longitudinal approach with a pre-pandemic baseline would have been more accurate in highlighting the detrimental effects of the restrictive measures due to the COVID-19 outbreak. However, the unexpected nature of the pandemic made such a study hardly feasible, and the matching of age and gender between the two samples should increase the reliability of our results. 

## 5. Conclusions

In light of our findings and considering the bidirectional relationship between sleep and mental health [75,76,77], we encourage paying special attention to university students during the current emergency period. Moreover, considering that the above-reported vulnerability of this population was also present before the COVID-19 outbreak, we suggest the implementation of preventive interventions focused on this specific group. This is of particular importance, as depression is associated with elevated probability of dropping out of the university [78], in addition to being related to suicidal ideation [79]. Indeed, the suicide rates showed an increase during the pandemic emergency among students [15]. Finally, supportive interventions could be particularly important as the detrimental impact of the COVID-19 pandemic on sleep and mental health seems to persist in the long run [60], in order to avoid the chronicity of such disorders among this at-risk population.

## Figures and Tables

**Table 1:** Mean ± standard deviation of questionnaire scores (PSQI, ISI, BDI-II), PSQI sub-components, bedtime, and get up time of the two groups (pre-pandemic, lockdown). Results of *t*-test comparisons are also reported (t_degrees of freedom_, *p*).

	Pre-Pandemic Mean ± Std. Dev.	Lockdown Mean ± Std. Dev.	t_478_	*p*
PSQI total score	5.96 ± 2.64	6.61 ± 2.92	−2.55	0.01
Subjective sleep quality	1.14 ± 0.59	1.34 ± 0.74	−3.34	<0.001
Sleep latency	1.54 ± 0.84	1.56 ± 0.97	−0.25	0.8
Sleep duration	0.48 ± 0.65	0.52 ± 0.69	−0.61	0.54
Habitual sleep efficiency	0.70 ± 0.88	0.55 ± 0.85	1.79	0.07
Sleep disturbance	1.33 ± 0.50	1.35 ± 0.57	−0.34	0.73
Sleep medications	0.10 ± 0.41	0.16 ± 0.57	−1.39	0.17
Daytime dysfunction	0.80 ± 0.70	1.21 ± 0.78	−4.8	<0.001
ISI total score	7.33 ± 4.4	8.34 ± 4.93	−2.35	0.02
BDI-II total score	10.17 ± 8.55	14.18 ± 8.97	−5.02	<0.001
Bedtime (hh:mm)	00:27 ± 01:15	00:52 ± 01:38	−3.11	0.002
Get up time (hh:mm)	09:26 ± 01:29	09:34 ± 01:33	−1.02	0.31

Abbreviations: PSQI, Pittsburgh Sleep Quality Index; ISI, Insomnia Severity Index; BDI-II, Beck Depression Inventory-Second Edition.

## Data Availability

The data presented in this study are available on request from the corresponding author.
